# Caffeine Intake and Mental Health in College Students

**DOI:** 10.7759/cureus.14313

**Published:** 2021-04-05

**Authors:** Raphael A. O Bertasi, Yasmine Humeda, Tais G. O Bertasi, Zoe Zins, Justin Kimsey, George Pujalte

**Affiliations:** 1 Department of Family Medicine, Mayo Clinic, Jacksonville, USA; 2 Department of Family Medicine, Florida State University College of Medicine, Tallahassee, USA

**Keywords:** college student, depression, caffeine, anxiety, coffee, mental health

## Abstract

Background

The effect of caffeine on the human body, both short-term and long-term, has been studied in great depth, particularly its association with psychiatric disorders. This study aims to investigate whether there is a correlation between caffeine intake and anxiety and depression among college students.

Methodology

A survey was administered to college students at Florida State University. Data regarding participant characteristics and caffeine intake were collected. Generalized Anxiety Disorder-7 and Patient Health Questionnaire-9 scores were used to assess symptoms of anxiety and depression, respectively.

Results

A total of 114 participants were included in the survey, consisting mainly of women (94 [82.5%]) and junior-level students (37 [32.5%]). The main source of caffeine was coffee (64.0%), and the main reasons for caffeine intake were pleasure (43.9%) and to study outside of class (29.8%); however, no association was found between sex or grade point average and number of cups of caffeine consumed. Upper levels of education (super senior or fifth-year students), depressive symptoms (poor appetite, overeating, sleep disorders, depressed mood), and anxiety were statistically associated with greater caffeine intake (*P *< 0.05).

Conclusions

As caffeine is commonly consumed and our study showed that its intake was associated with depressive symptoms and higher levels of anxiety in college students, further studies are needed to determine a possible causality, so that measures may be taken to educate these students about alternative methods for increasing energy and alertness.

## Introduction

Due to its availability, caffeine is widely used as a source of energy. Coffee, pills, soda, and energy drinks are some of the most popular sources of caffeine. Some of the benefits that have been attributed to moderate caffeine intake include increased attention, alertness, mood elevation, increased cognitive function and fewer cognitive failures, lower risk of suicide, and fewer depressive symptoms [1]. Thus, caffeine use is extremely prevalent among college students. The college lifestyle, however, appears to lend itself to higher caffeine intake compared to the rest of the population [2]. College students use very high doses of caffeine, an average of over 800 mg/day, which is approximately double the recommended safe dosage [3].

The short-term and long-term effects of caffeine on the human body have been studied. Research to date has primarily focused on caffeine’s exacerbation of anxiety, sleep disorders, and depression in patients diagnosed with psychiatric symptoms [4-6]. Caffeine consumption has been associated with an increase in anxiety in adults with generalized anxiety disorder [7]. However, those who consume caffeine also tend to experience greater positive effects on behavior, including alertness and arousal [5].

This study examines whether there is a correlation between caffeine intake and anxiety and depression in college students (both with and without a previous diagnosis of either mental health condition).

## Materials and methods

An online survey using Qualtrics XM (Qualtrics, Seattle, WA, USA) was randomly distributed to college students at Florida State University via email, Twitter, and Facebook in 2016. Participants were eligible if they were college students enrolled at Florida State University and could answer a questionnaire via email. Students who for any reason were not able to answer questions on an emailed questionnaire were excluded. We collected demographic characteristics (e.g., sex and year in school), grade point average (GPA), previous diagnosis of depression or anxiety, and caffeine intake-related information (e.g., source, frequency, and reason). For this study, one cup was defined as eight ounces of liquid. The Generalized Anxiety Disorder-7 (GAD-7) [8] and Patient Health Questionnaire-9 (PHQ-9) [9] were included in the survey to assess anxiety and depressive diagnoses, respectively. The sensitivity and specificity of GAD-7 and PHQ-9 diagnosis, using a cut point of 10, are 89% and 88% and 82% and 88%, respectively [8,9]. Therefore, a diagnosis of anxiety and depression were considered when the participant reached a score greater than or equal to 10 on GAD-7 and PHQ-9, respectively. However, when the score was <10, we considered the greater the total score on each questionnaire the higher the level of each disorder.

Questions were in the English language with multiple-choice answers and were filled by the participants. The questions applied in the survey are provided in the Appendix. Data were anonymously extracted to an Excel spreadsheet (Microsoft, Inc., Redmond, WA, USA).

Each question had four possible responses: zero = “not at all,” one = “several days,” two = “over half the days,” and three = “nearly every day.” The total scale score classified the level of anxiety and depression, with higher scores representing a greater severity of either disorder.

Each questionnaire answer was evaluated individually and as part of the total score of the complete anxiety and depression assessment. Statistical analysis was performed with SPSS, version 21 (IBM Corp., Armonk, NY, USA). As the data were not normally distributed, the Kruskal-Wallis test was used to compare numeric and categorical data, and in case of statistically significant results, the Mann-Whitney test was performed. The Pearson product-moment correlation coefficient (r) assessed a correlation between the two numeric datasets. P values less than 0.05 were considered statistically significant.

This study was approved by the Mayo Clinic Institutional Review Board (#16-005552). Online informed consent was obtained from all participants before collecting any data.

## Results

The survey was answered by 114 participants, including 94 women (82.5%) and 20 men (17.5%). Most of the students were in their junior year of college (37 [32.5%]) and had a GPA between 3.5 and 4.0 (75 [65.8%]). Their main source of caffeine was coffee (73 [64.0%]), and their main reasons for caffeine intake were pleasure or enjoyment (50 [43.9%]) and to study outside of class (34 [29.8%]). Table 1 presents all demographic and caffeine-related characteristics of the participants.

**Table 1:** Demographic characteristics of the study participants.

Characteristic	Total (N = 114)
School year, No. (%)	
Freshman	12 (10.5)
Sophomore	26 (22.8)
Junior	37 (32.5)
Senior	35 (30.7)
Super senior or fifth year	4 (3.5)
Sex, No. (%)	
Female	94 (82.5)
Male	20 (17.5)
Previous diagnosis of depression or anxiety, No. (%)	
Never	79 (69.3)
Both	16 (14.0)
Anxiety	15 (13.2)
Depression	2 (1.8)
Missing	2 (1.8)
Source of caffeine, No. (%)	
Coffee	73 (64.0)
Tea	17 (14.9)
Soda	12 (10.5)
Other	9 (7.9)
Missing	3 (2.6)
Reason for caffeine consumption, No. (%)	
To stay awake in class	14 (12.3)
Studying/homework (e.g., outside of class)	34 (29.8)
For pleasure or enjoyment	50 (43.9)
Other	14 (12.3)
Missing	2 (1.8)
Grade point average, No. (%)	
<3.0	8 (7.0)
3.0-3.4	28 (24.6)
3.5-4.0	75 (65.8)
Missing	3 (2.6)

Students’ year at school and their caffeine source were associated with the number of cups of caffeine they consumed per week (P = 0.02 and P < 0.001, respectively). Super senior students consumed more caffeine than freshmen, sophomores, and juniors (P = 0.02, P = 0.02, and P = 0.05, respectively), while juniors consumed more coffee than freshmen (P = 0.03). Moreover, coffee and soda were used as caffeine sources more often than tea (P < 0.001 and P = 0.01, respectively). There was no association between sex or GPA and the number of cups of caffeine consumed.

**Figure 1:**
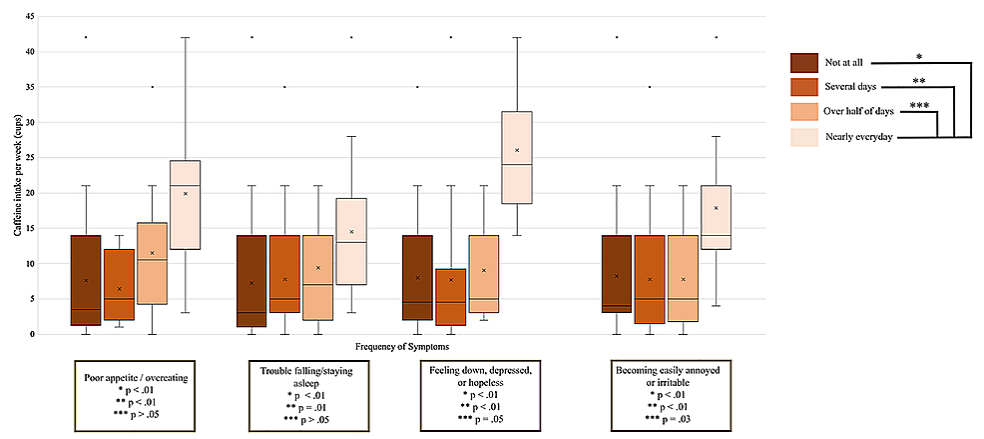
Questionnaire questions associated with caffeine intake per week. Box plot comparing amount of caffeine intake per week and frequency of symptoms identified in the Generalized Anxiety Disorder-7 and Patient Health Questionnaire-9 questionnaires with P < 0.05 in the Kruskal-Wallis test (P values for the Mann-Whitney test assessing each answer are also in the graph). One cup equals eight ounces of liquid.

Regardless of previous diagnoses of anxiety or depression, one item from the GAD-7 and three from the PHQ-9 were significantly associated with caffeine consumption (P < 0.03). The items listed were “poor appetite or overeating” (PHQ-9), “trouble falling or staying asleep or sleeping too much” (PHQ-9), “feeling down, depressed, or hopeless” (PHQ-9), and “becoming easily annoyed or irritable” (GAD-7). Students who experienced any of the above problems “nearly every day” had more caffeine intake per week than those who answered “not at all” or “several days” (P < 0.05; Figure 1).

**Figure 2:**
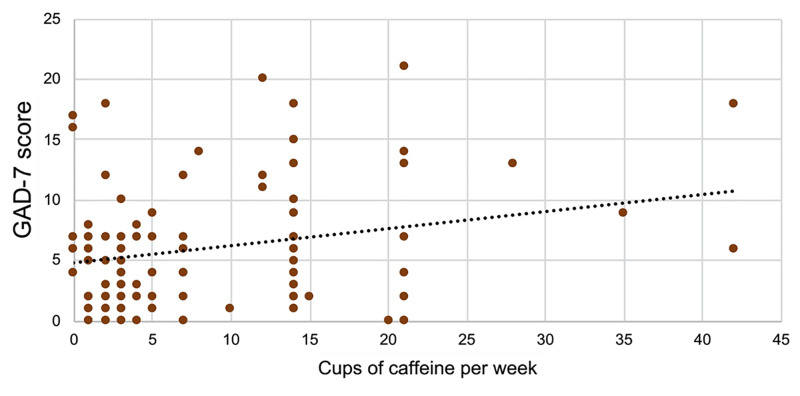
Correlation between total score of GAD-7 and caffeine intake. GAD-7 = Generalized Anxiety Disorder-7 One cup equals eight ounces of liquid Correlation: r = 0.240, P = 0.01.

Moreover, there was a positive correlation between the GAD-7 anxiety score and the number of cups of caffeine consumed per week (r = 0.24, P = 0.01), but not with the depression score on the PHQ-9 (r = 0.01, P = 0.88; Figure 2).

For participants without a previous diagnosis of anxiety and depression, only the question regarding “poor appetite or overeating” had a significant difference: those who answered “nearly every day” or “over half of days” had more caffeine intake per week than those who answered “not at all” or “several days” (P < 0.05). A slightly positive, but not statistically significant association, was found between caffeine intake and scores greater than or equal to 10 in GAD-7 and PHQ-9 (P = 0.09 and P = 0.29, respectively).

## Discussion

The results of this study suggest a correlation between high caffeine intake and symptoms of anxiety and depression in college students. Caffeine is the most commonly consumed central nervous system stimulant worldwide [10], with coffee being the most preferred source [11]. Tea, soda, chocolate, and energy drinks are also commonly used by people of all ages, with some containing even higher amounts of caffeine [12]. The main motivations for caffeine consumption include enhanced physical performance, greater energy, personal enjoyment, improved concentration, reduced stress, and fulfilling social purposes [13,14].

Mahoney et al. [15] showed that, among 1,145 college students, the main motivation for caffeine consumption was increased wakefulness, followed by taste. Meanwhile, Micoulaud-Franchi et al. [16] found that enhancement of academic performance and improvement of wakefulness among college students were the main reasons for caffeine consumption. These studies are partially in line with our own, which reported enjoyment and optimized studying as the primary motivations.

The motives for caffeine consumption, and the association found between upper levels of education (i.e., super senior) and greater caffeine intake, suggest that students might believe caffeine may help improve academic performance. Interestingly, educational achievement was shown to have a negative association with caffeine intake [2]. This finding was not contradicted by our study, which showed no positive association between caffeine intake and GPA. The sought-after effect of caffeine on enhanced academic performance deserves further investigation.

Behavior and mood symptoms linked to psychiatric disorders have also been associated with caffeine consumption. Caffeine inhibits adenosine receptors in the central nervous system, mainly in the hippocampus, amygdala, and prefrontal cortex (locations with high concentrations of these receptors that are associated with emotion, cognition, and motivation), which might play a role in the association between depression and caffeine consumption [17-19].

Several studies have reported an inverse association between depression and caffeine intake, which suggests that caffeine consumption may work as a protective factor for depression [19-22]. However, it is important to note that these studies included only older participants, with mean ages greater than 40. A systematic review of 15 articles assessing people of all ages found that a high consumption of coffee decreases the risk of depression [23]. However, if only children and adolescent studies from the systematic review are considered, this association ceases to exist. Studies that assessed children and adolescents showed only a positive association between depression and caffeine intake [24-26]. Iranpour and Sabour [19] showed that an increment of 1 mg of caffeine per day had a different effect on depressive symptoms in each age range; however, further studies should be conducted to clarify such age-related effects of caffeine intake.

There is still no clear evidence to support the idea that caffeine causes depression. Either people prone to depression self-medicate with more caffeine to improve their energy and concentration, or caffeine properties interact in the brain, leading to depressive symptoms [27]. We hypothesize that the age of consumption may have an important effect on this association. In our study of college students, we found that symptoms of depression such as poor appetite or overeating, sleep disturbances, and feelings of hopelessness were positively associated with caffeine consumption (Figure 1).

Similarly, caffeine may affect anxiety-like behaviors by inhibiting adenosine receptors, particularly the A2A receptor [28]. Higher levels of caffeine intake have been linked to higher anxiety levels when consuming at least one cup of coffee per day [17,24,29]. Indeed, in our study, participants with higher levels of caffeine intake had higher GAD-7 scores (Figure 2). It is worth noting that there are some genetic variations in adenosine receptors that lead to different anxiety behaviors in light caffeine users [30]. Therefore, levels of caffeine sensitivity may vary in this population and should be addressed in further studies.

Our study has some limitations, such as its design and small sample size. Although interesting, our findings are speculative. They should not be extrapolated and must be analyzed with caution. However, our results do contribute to the understanding of the effects of caffeine on psychiatric disorders, especially in young adults/adolescents, as there are scant data in this age range. Additional studies should be performed on the physical and psychological repercussions of caffeine on people of different ages, especially youth.

## Conclusions

As our study showed an association between caffeine intake and depressive symptoms, caffeine cannot be deemed a protective factor for depression as it is in adults older than 40. Additionally, greater consumption of caffeine by college students was associated with higher levels of anxiety, as measured by the GAD-7 scores. As caffeine is commonly consumed and our study showed that its intake was associated with depressive symptoms and higher levels of anxiety in college students, further studies are needed to determine a possible causality, so that measures might be taken to educate these students about alternative methods for increasing energy and alertness.

## Appendices

**Table 2:** Survey questions.

General questions (1 cup = 8 oz of liquid)
∙ Year in school? (Freshman, Sophomore, Junior, Senior, Fifth Year or “Super Senior”)
∙ Sex (Male, Female)
∙ Have you been diagnosed with anxiety or depression? (Anxiety, Depression, Both, Never)
∙ What is your current GPA? (<2.0, 2.0-2.4, 2.5-2.9, 3.0-3.4, 3.5-4.0)
∙ How much caffeine do you consume per day? (None, 1, 2, 3, 4, 5, 6, >6 cups)
∙ How many times a week do you consume caffeine? (Never, 1, 2, 3, 4, 5, 6 times/week, Daily)
∙ What source of caffeine do you utilize most often? (Coffee, Tea, Soda, Other)
∙ For what reason do you most often consume caffeine? (Studying/Homework, To stay awake in class, For pleasure or enjoyment, e.g., You like the taste of coffee or tea, Other)
Patient Health Questionnaire-9 (PHQ-9)
Over the past 2 weeks, how often have you been bothered by any of the following problems? (Not at all, Several days, More than half the days, or Nearly every day)
∙ Little interest or pleasure in doing things
∙ Feeling down, depressed, or hopeless
∙ Trouble falling or staying asleep, or sleeping too much
∙ Feeling tired or having little energy
∙ Poor appetite or overeating
∙ Feeling bad about yourself, or that you are a failure or have let yourself or your family down
∙ Trouble concentrating on things, such as reading the newspaper or watching television
∙ Moving or speaking so slowly that other people could have noticed. Or the opposite—being so fidgety or restless that you have been moving around a lot more than usual
∙ Thoughts that you would be better off dead, or of hurting yourself in some way
Generalized Anxiety Disorder-7 (GAD-7)
Over the past 2 weeks, how often have you been bothered by any of the following problems? (Not at all, Several days, More than half the days or Nearly every day)
∙ Feeling nervous, anxious, or on edge
∙ Not being able to stop or control worrying
∙ Worrying too much about different things
∙ Trouble relaxing
∙ Being so restless that it’s hard to sit still
∙ Becoming easily annoyed or irritable
∙ Feeling afraid as if something awful might happen
